# Second-harmonic generation microscopy of murine scleral remodeling by collagenase and reparative collagen mimetic peptides

**DOI:** 10.3389/fmed.2025.1514073

**Published:** 2025-05-20

**Authors:** Daniel E. Savage, Aldo Tecse, Jiaxi Zhou, James A. Germann, Mark R. Buckley, Robert O. Baratta, Brian J. Del Buono, Eric Schlumpf, Michael Telias, Susana Marcos

**Affiliations:** ^1^John A. Moran Eye Center, University of Utah, Salt Lake City, UT, United States; ^2^Flaum Eye Institute, University of Rochester, Rochester, NY, United States; ^3^Department of Biomedical Engineering, University of Rochester, Rochester, NY, United States; ^4^Center for Visual Science, University of Rochester, Rochester, NY, United States; ^5^The Institute of Optics, University of Rochester, Rochester, NY, United States

**Keywords:** myopia, collagen, sclera, collagen mimetic peptides, second-harmonic generation

## Abstract

**Introduction:**

Myopia, resulting from an excessive axial elongation of the eye, is increasing worldwide at alarming rates. This investigation is a pilot study to determine if a novel collagen mimetic peptide (CMP) has a reparative function for scleral collagen organization after collagenase digestion, a cause for scleral thinning and increased creep rates, as this may have application in the pharmacologic treatment of myopia.

**Methods:**

Fresh, *ex vivo*, scleral tissue samples from 3 albino Sprague–Dawley rats (5 eyes) and from 8 C57/Black mice (8 eyes) underwent sequential collagenase digestion and treatment with a CMP solution. Full-thickness second-harmonic generation (SHG) microscopy was performed over a 200 μm × 200 μm area through depth on each of the untreated samples (either scleral tissue samples or full intact eyes), and again after each sequential treatment. The organization of the collagen fibers at each tissue depth was quantified using a previously validated order coefficient (OC). This measure of collagen organization was then used to compare between the untreated, collagenase-digested, and CMP-treated tissue.

**Results:**

SHG microscopy of the untreated scleral tissue showed a high degree of organization. Collagenase treatment resulted in a subjective straightening of the collagen fibers and a widening of the inter-fiber spacing with a statistically significant reduction of the OC (*p* < 0.05). CMP treatment of digested sclera resulted in a collagen organization that was more similar (i.e., not significantly different) from untreated tissue at depths up to 60 μm (*p* < 0.05). The restoration of collagen organization was found both in the treated excised rat scleral samples (OC: 0.30 ± 0.01 normal tissue, 0.37 ± 0.05 collagenase-digested and 0.28 ± 0.03 CMP-treated until 20 μm) and on intact mice eyes (OC: 0.25 ± 0.01 normal tissue, 0.30 ± 0.05 collagenase-digested and 0.24 ± 0.01 CMP-treated).

**Discussion:**

CMP treatment induced scleral collagen reorganization after collagenase digestion in murine models. These effects are consistent with inhibition or reversal of collagen enzymatic digestion. These results suggest that specific CMPs may have utility in the treatment of progressive myopia.

## 1 Introduction

To address the visual complications associated with myopia and prevent its advancement into severe forms, there has been a growing interest in innovative therapeutic approaches. Myopia has emerged as a global health concern with a rising prevalence, presenting significant challenges to public healthcare systems (1). Progressive myopia, especially high myopia, carries an elevated risk of ocular complications, including retinal detachment, myopic maculopathy, and glaucoma, all of which can ultimately lead to vision impairment and blindness (2, 3).

Collagen, serving as the primary structural component of the sclera, plays a pivotal role in upholding the biomechanical integrity of the eye (4–6). Studies have shown an association between myopia and increased protease activity within the sclera (7–10). Consequently, interventions capable of modulating enzymatic collagen degradation within the sclera may hold promise as potential therapeutic avenues for myopia. Among these interventions, collagen mimetic peptides (CMPs) have emerged as potential agents for repairing and restoring collagen organization and structure (11, 12).

CMPs are synthetic peptides that mimic the structural and functional properties of natural collagen. CMPs are typically composed of amino acid sequences that resemble the repeating Gly-X-Y motif found in natural collagen, where Gly is glycine and X and Y can be any amino acid (typically proline or substituted proline residues) (13). CMPs have previously been used in tissue engineering and regenerative medicine applications to facilitate tissue repair and regeneration (13–15). The CMP investigated in the present study was similarly designed to attempt to repair helical collagen damaged by proteases, thus restoring the structural and functional properties of collagen to that seen in its undamaged state.

In this investigation, we employed second-harmonic generation (SHG) microscopy to evaluate how a novel CMP affects collagen in murine scleral tissue after collagenase treatment (16). Our primary objective was to assess the ability of this novel CMP to restore the organization of collagen that has undergone enzymatic digestion within rat scleral tissue. If successful, this endeavor could lead to innovative therapeutic approaches aimed at slowing the progression of myopia.

## 2 Materials and methods

### 2.1 Murine samples

Scleral tissue from albino Sprague–Dawley rats (Charles River Laboratories, Inc., Wilmington, MA, United States) and male C57BL/6 J mice (Jackson Laboratory, strain #000664) were used as the scleral collagen model. All animal use in this study conformed to the standards set in the Association for Research in Vision and Ophthalmology (ARVO) Statement for the Use of Animals in Ophthalmic and Vision Research and was approved by the University of Rochester University Committee on Animal Resources. The minimum number of samples deemed necessary for this pilot study were used in order to minimize animal sacrifice.

Fresh scleral tissue was obtained from 3 albino Sprague–Dawley rats euthanized by KCl injection. The 6 rat eyes were dissected, and the extra-scleral tissue, such as connective and muscle tissue, was removed using pulling it against the grain with angled forceps (17). Mice eyes (*n* = 8) were obtained postmortem (P28-P49) following euthanasia by isoflurane overdose followed by cervical dislocation. The extra-scleral tissue was then meticulously removed similarly to the procedure for rat sclerae (17). The samples were preserved in phosphate-buffered saline (PBS) at 4°C when not in use and all experiments were done less than 12 h after euthanasia.

### 2.2 Imaging protocol

**Figure 1:**
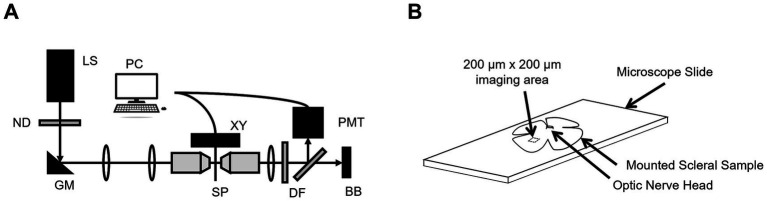
**(A)** The custom-built SHG microscope where LS = laser source, ND = neutral density, GM = galvanometer mirrors, PC = computer, XYZ = translation stage, SP = sample plane, DF = dichroic filters, PMT = photomultiplier tube, and BB = beam block. **(B)** A representative anatomical sketch of a scleral sample mounted on a microscope slide with the optic nerve head and SHG collection area illustrated (not to scale).

A custom-built SHG microscope was used to image the scleral tissue samples. The system was a new generation of a system previously described redundant (18), with some new features, including the illumination laser. Briefly, a femtosecond laser source (Fyla, Spain, 950–1150 nm, 20 fs, 80 MHz) was attenuated to 28.5 mW average power and focused into the sample plane using a high numerical aperture water-immersion microscope objective. The sample plane was mounted on an automated 3-axis XYZ translation stage. The backscattering signal (λ_0_ = 550 nm BW = 200 nm) from the sample plane was de-scanned by the same objective and focused onto a photomultiplier tube (Figure 1A). Calibrations of the system were performed using tissue from a single rat eye.

Each rat (*n* = 5) scleral tissue sample had radial cuts made into it to allow it to be mounted flat on a standard microscope slide with a drop of a balanced saline solution (BSS) and a coverslip. Then, a series of 200 μm × 200 μm en face images (Lateral resolution: 0.375 μm) were taken at 5 μm intervals through the full thicknesses of the sclerae approximately 2 mm from the optic nerve (Figure 1B).

**Figure 2:**
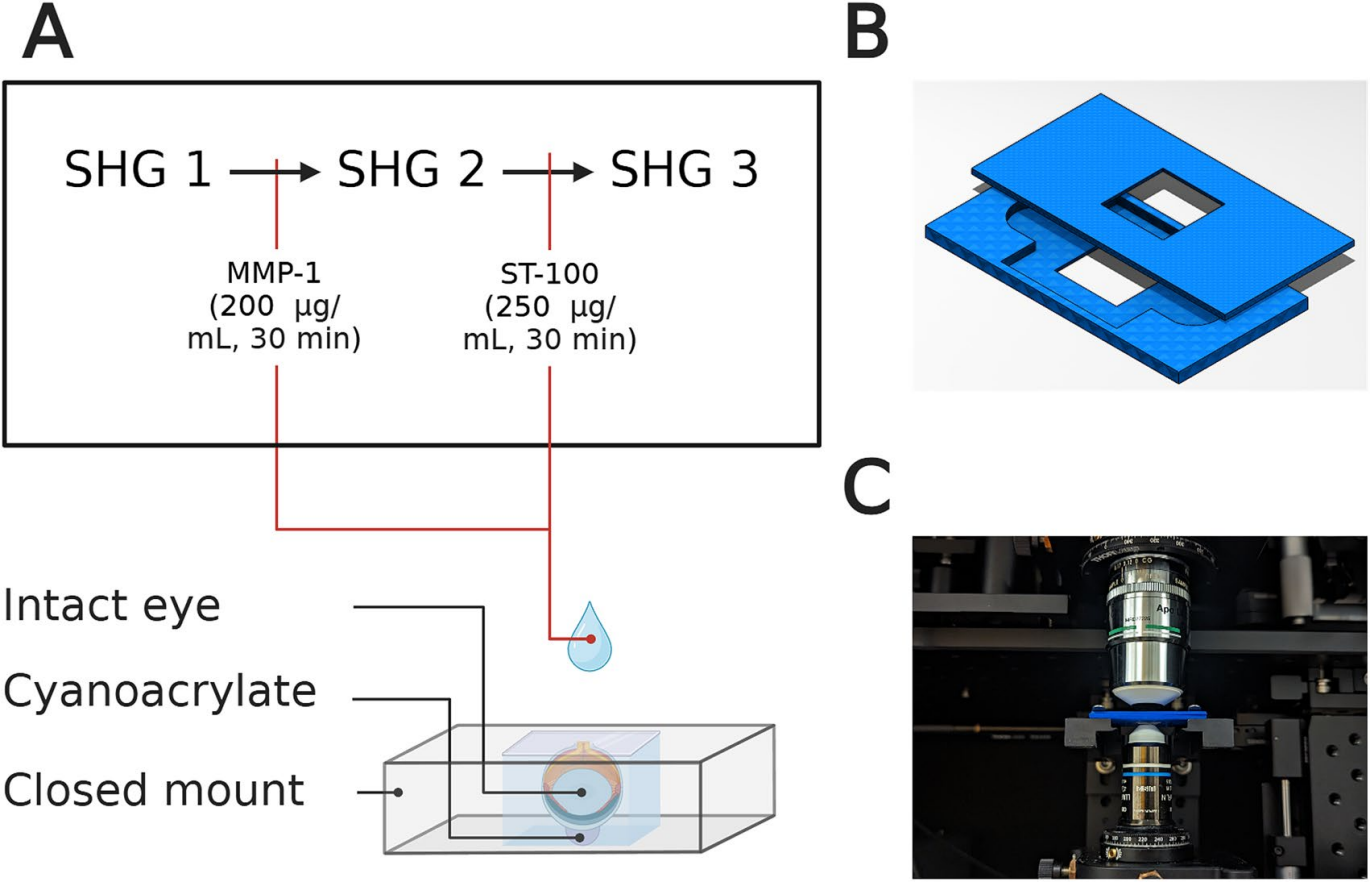
**(A)** Representative illustration of whole-eye setup for SHG imaging for untreated sample (SHG 1), after collagenase (MMP-1) treatment (SHG 2), and after CMP treatment (SHG 3). The cornea is fixed at the bottom using cyanoacrylate and the eye is immersed in BSS during imaging. **(B)** Top and bottom pieces of the intact-eye mount that were 3D-printed for each sample. **(C)** Representative image of the intact-eye mount with the custom-built confocal microscope before imaging.

For intact eye scleral imaging in mice, a compressive fixture was used to fix the cornea at base with cyanoacrylate, and to allow whole eye immersion in BSS (Figure 2A). The fixture consisted of two 3D printed pieces leaving a cuboidal space with a height of 3 mm (Figure 2B). This design enabled intact-eye imaging of the scleral tissue at the top (Figure 2C). Then, a series of 200 μm × 200 μm en face images (Lateral resolution: 0.375 μm) were acquired through the full thickness at 2-μm intervals, and measured at 1 mm away from the optic nerve head.

### 2.3 Tissue treatment

After baseline SHG imaging, the tissue samples were partially digested on microscope slide (excised samples) or in the fixture (whole eyes) using a 200 μg/mL solution of recombinant collagenase derived from *Clostridium histolyticum* with recombinant thermolysin (TDzyme^®^ C/T; TDzyme, Inc., Dong-gu, Daegu, Republic of Korea) for 30 min. This was accomplished by removing the coverslip and placing 1 to 2 drops of the solution onto the surface of the tissue sample, enough to completely coat the apical surface of the sample. The samples were subsequently rinsed with BSS and re-imaged at approximately the same locations. The digested samples were then bathed in a 294 nM CMP solution (Stuart Therapeutics, Inc., Stuart, FL, United States) for 30 min again using 1 to 2 drops on the top surface of the tissue sample. The CMP solution “ST-100” provided by Stuart Therapeutics was composed of a linear, single-chain 21-mer collagen mimetic sequence with a (Gly-Pro-Pro)7 motif. Lastly, the tissue samples were rinsed with BSS and underwent final SHG microscopy. It is important to note that all tissue treatments were performed without removing the tissue sample from the microscope slide or fixture, thus allowing for reasonable co-localization of pre-and post-treatment SHG images.

### 2.4 Image analysis

After collection, the SHG image stacks from each sample were analyzed using Matlab (MathWorks, Inc., Portola Valley, CA, United States). Two algorithms were used to estimate the collagen bundles orientation. Preprocessing of images for both algorithms included median filter for removing Poisson noise typical of SHG and edge sharpener based on a Gaussian Low-pass filter (N = 10, SD = 10) was to use to enhance high spatial frequency contrast.

In the first algorithm, a two-dimensional Fourier algorithm was used to analyze the whole en face image and a Hanning window was used to remove the edge effect of image cropping. Then, the higher energy density peaks were selected as these represent more collagen bundles at a particular angle (Supplementary Figure S1). The angles at which these peaks are present were stored.

In the second algorithm, a gradient pyramid of the whole image is computed, and no windowing is required given that this algorithm does not require a spatial frequency analysis. Then, Principal Component Analysis (PCA) is used to estimate the local orientation for each overlapping square windows (sides of 1.88 μm). Assuming a constant orientation inside the windows, the gradient vector should be orthogonal to the orientation of the image pattern. So, an optimization task was defined to find the vector that maximizes orthogonality (19). Then, an intensity mask was used to preserve orientation information only where collagen signal was detected (Supplementary Figure S2) (20). Finally, the orientation information can be displayed spatially by color coding, with the intensity representing the presence of collagen at that particular depth (20, 21).

A previously validated metric, the order coefficient (OC), was used to quantify the collagen arrangement based on the orientation provided by each algorithm through depth. The OC represents a measurement of anisotropy of the scleral collagen fibers which ranges from 0 to 1. Increasing magnitude of the OC indicates increasing uniformity in the direction collagen fibers run in each SHG image. Collagen orientation information is transformed into polar coordinates and its distribution is analyzed using angular windows of 7.5° with shifts of 1° (22). A control test on 5 samples and 3 simulated fiber distributions demonstrated the equivalency between the two described algorithms in the estimation of OC.

### 2.5 Statistical analysis

The OC for each of the rat samples was calculated at each depth position then averaged across all 5 samples over sequential 20 μm depth intervals. This 20 μm depth interval average OC was statistically compared using a paired Student’s two-tail t-test between the tissue samples in in different experimental states: the untreated and post-collagenase states, the untreated and post-CMP treatment states, and the post-collagenase and post-CMP treatment states. A paired test was utilized as data was comprised of measurements taken on a given sample before and after the various interventions and thus were treated as dependent variables in hypothesis testing using Excel (Microsoft, Inc., Redmond, WA, United States). The OC for each of the mice eyes was calculated over their entire depth, around 20 μm thick (23), considering a single value average over the whole sample depth. One-way analysis of variance (ANOVA) with *post hoc* Tukey–Kramer analysis was used to test statistical significance in OC across treatments.

## 3 Results

**Figure 3:**
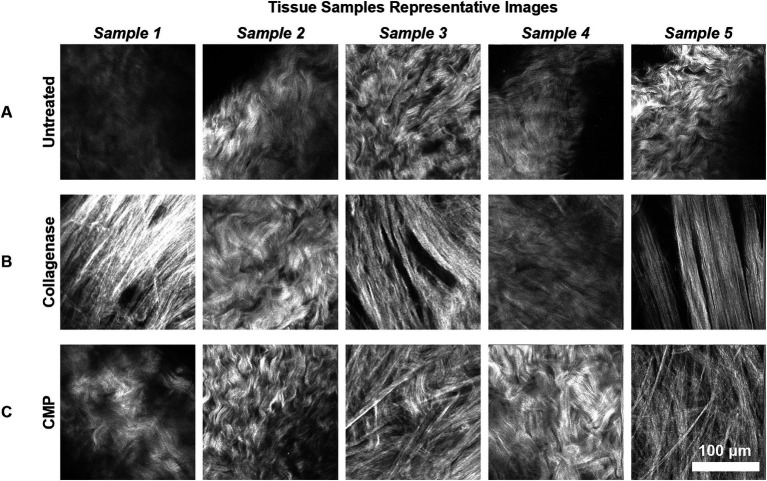
Representative 200 μm × 200 μm SHG images of the rat sclerae tissue samples are shown. The images are taken from mid-depth points of each sample (mean depth = 32.5 μm). **(A)** The untreated rat sclera samples. **(B)** Samples from **(A)** after subsequent collagenase digestion. **(C)** Samples from **(B)** after subsequent CMP treatment.

Subjectively, SHG microscopy of the untreated scleral tissue revealed a high degree of collagen fiber curling and inter-fiber connectedness (Figure 3A). After collagenase treatment, the collagen fibers assumed a straightened configuration with widening of the inter-fiber spacing (Figure 3B). Tissue samples imaged after treatment with both collagenase and the CMP solution contained collagen fibers assuming a more interconnected and curled appearance compared to the collagenase treated tissue (Figure 3C).

**Figure 4:**
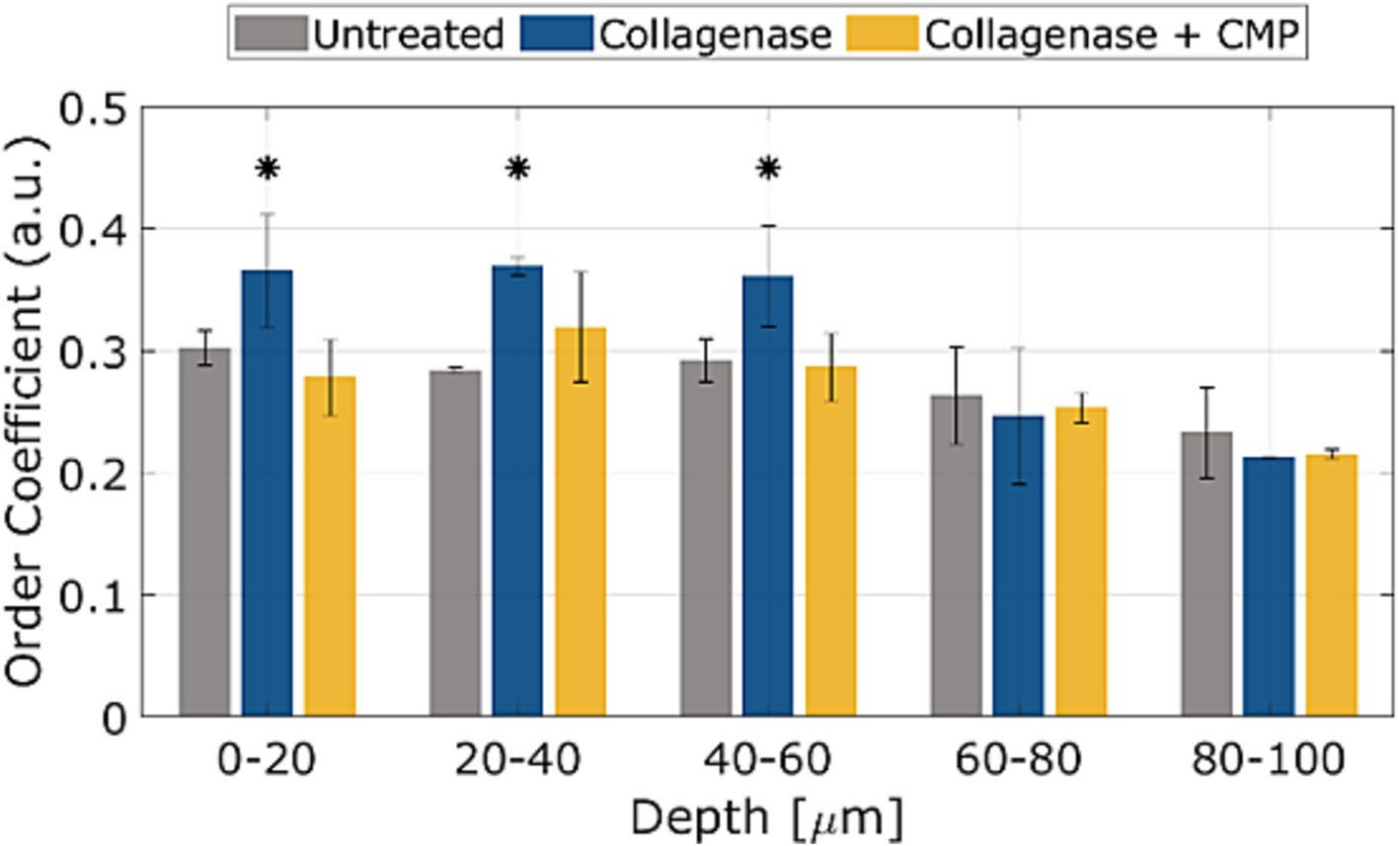
OCs as a function of depth for untreated, post-collagenase treated, and post-collagenase- and CMP-treated tissue sample data. The OCs shown are averaged across all 5 samples for each depth position, and then averaged over the depth values in each range. Error bars represent the standard deviation. Asterisks indicate statistical significance (*p* < 0.05) between the untreated and collagenase groups and between the collagenase and collagenase + CMP groups using a paired student’s two-tail *t*-test. No statistically significant difference in the average OC was noted between the untreated and collagenase + CMP groups.

**Figure 5:**
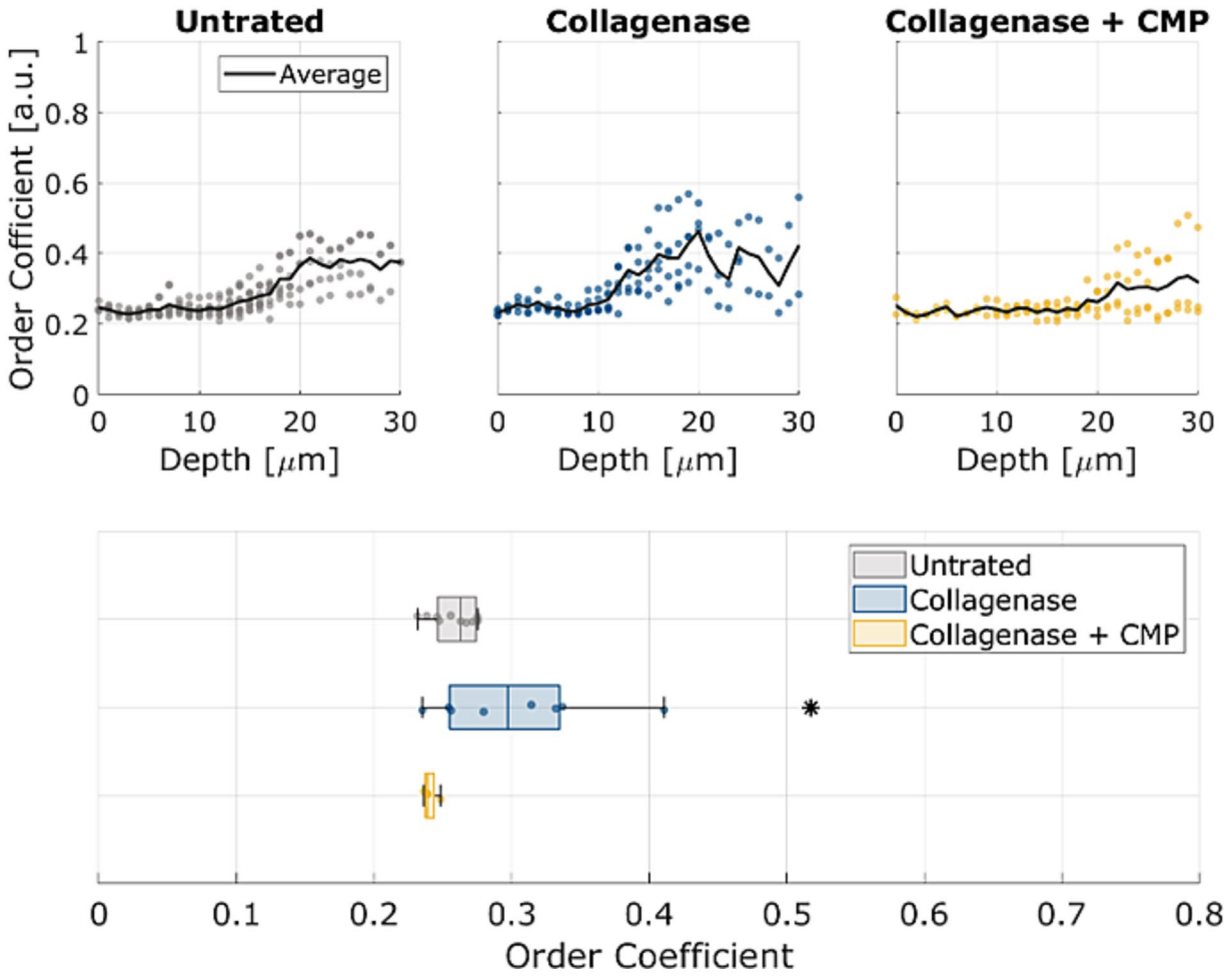
OCs as a function of depth for untreated, post-collagenase treated, and post-collagenase- and CMP-treated tissue sample data. The black line represents the average OC across all 8 samples through depth for each treatment. At the bottom, standard boxplots based on the average through the whole depth are shown. The asterisk indicates statistical significance (*p* < 0.05) between the untreated and collagenase groups and between the collagenase and collagenase + CMP groups using ANOVA and a *post hoc* Tukey–Kramer analysis.

The average OC in rat sclerae (from excised samples) across all 5 samples as a function of depth within the tissue samples is shown in Figure 4. The numerical OC and corresponding *p*-values are listed in Table 1. Collagenase digestion of the tissue samples resulted in a statistically significant increase in the OC compared to the untreated tissue (*p* < 0.05). CMP treatment of digested sclera resulted in a collagen organization that was more similar (i.e., not significantly different, *p* > 0.05) from untreated tissue at all depths. The averages OC in mice sclerae (from whole eyes) along the sample depth, across all 8 eyes for the different treatments (Figure 5). Similarly, to experiment in rat sclerae, MMP-1 (0.30 ± 0.05) increased the alignment of collagen bundles (*p* < 0.05) with respect to the virgin state (0.25 ± 0.01) in mice sclera. Also in this group, CMP ST-100 induced a significantly reduction (*p* < 0.05) of the OC (0.24 ± 0.01) (Figure 5).

**Table 1:** The average OCs across all 5 samples as a function of depth (*N* = 20 per depth interval) for untreated, post-collagenase treated, and post-collagenase- and CMP-treated tissue samples with standard deviations and *p*-values using a paired student’s two-tail *t*-test.

Depth [\mu m]	Average order coefficient						Paired *t*-test-value		
	Untreated	SD	Collagenase	SD	CMP	SD	Untreated vs. collagenase	Collagenase vs. CMP	Untreated vs. CMP
0–20	0.302	0.014	0.365	0.046	0.278	0.031	0.020*	0.006*	0.060
20–40	0.284	0.002	0.369	0.007	0.319	0.045	0.005*	0.018*	0.074
40–60	0.292	0.018	0.361	0.041	0.286	0.028	0.003*	0.013*	0.782
60–80	0.263	0.040	0.246	0.056	0.253	0.012	0.273	0.670	0.228
80–100	0.232	0.037	0.212	0.001	0.215	0.004	0.233	0.651	0.095

SD, standard deviation; CMP, Collagen Mimetic Peptide, *statistical significance (*p* < 0.05).

## 4 Discussion

The present study used SHG microscopy to investigate the potential of a novel CMP (ST-100) with a (Gly-Pro-Pro)7 motif to promote reparative changes in collagen organization within murine scleral tissue following collagenase digestion. The findings of this investigation are an important first step in elucidating a potential role for CMPs in addressing collagen degradation in the context of progressive myopia.

The effects of collagenase digestion on collagen fiber organization that we observed are consistent with the hypothesis that enzymatic degradation of the scleral extracellular matrix may play a role in the pathophysiology of progressive myopia (8). As noted, the untreated scleral tissue, as observed in our SHG microscopy images, exhibited a high degree of collagen fiber organization characterized by the typical curled appearance of scleral collagen fibers (24). However, collagenase treatment induced statistically significant changes in the collagen architecture consistent with a loss of collagen fiber interconnectedness, including straightening of the fibers and widening of the inter-fiber distances. These morphological changes may indicate a loss of scleral extracellular matrix strength and rigidity and may enable the elongation of the eye seen in progressive myopia (25–27).

Intriguingly, the application of CMP to the collagenase-digested scleral tissue samples appeared to have a reparative effect on collagen organization. The SHG microscopy images of tissue samples treated first with collagenase and then with CMP showed collagen fibers exhibited a more interconnected and curled appearance, which was visually distinct from the tissue after collagenase-only treatment. More importantly, the quantitative analysis using the OC revealed that CMP treatment of collagenase-treated tissue restored collagen organization to a state that was not statistically different from the untreated tissue at depths up to 60 μm in rat eyes, and over the full depth in mouse eyes. This finding suggests that CMPs have some potential to reverse the enzymatic degradation of collagen induced by collagenase, ultimately restoring the native collagen structure. It is possible that the CMPs may also have an inhibitory effect on the collagenase, however the present experiment was not designed to elucidate this.

Given that myopia is a progressive disease, further understanding of the mechanism by which CMPs impart their reparative effect is important for determining how effective CMP treatment may be over time. At least one other study has shown that CMPs are degraded by collagenase at a similar rate compared to natural collagen (28). Thus, the CMPs may act as a competitive antagonist to the collagenase enzyme, essentially preventing some quantity of the collagenase from binding to the scleral collagen. Alternatively, the CMPs may be intercalating into digested strands of damaged scleral collagen, effectively increasing crosslinking and fiber interconnectedness (29). A mixed mechanism is also possible. This study was not designed to elucidate the CMP reparative mechanism, but because it may inform the treatment potential of CMPs, it will be a subject of future investigations.

It is important to acknowledge that this study has certain limitations, including a small sample size, a study design that does not allow for differentiating the mechanism of the CMPs (i.e., enzymatic inhibition of the collagenase versus direct repair of the digested collagen fibers), and the use of *ex vivo* tissue. Further research is warranted to validate these preliminary results *in vivo* and to explore the long-term effects of CMP treatment on myopia progression and associated ocular complications. The experiment on intact mice eyes demonstrates that the finding holds when imaging is performed in a physiological setting more similar to *in vivo* conditions and in a backscattering mode. Additional next steps include studying the biomechanical properties of CMP-treated collagen to determine if there is a return to untreated levels of crosslinking and tensile strength.

In conclusion, this study provides initial evidence that CMP treatment can effectively restore collagen organization in collagenase-digested rat and mice scleral tissue. These findings suggest a potential role for CMPs in the development of innovative therapeutic strategies for the management of progressive myopia. Further research is needed to elucidate the precise mechanisms involved and to evaluate the clinical applicability of CMP-based interventions in myopia management.

## Data Availability

The raw data supporting the conclusions of this article will be made available by the authors, without undue reservation.
