# Supplementary figures and images for: Second-harmonic generation microscopy of murine scleral remodeling by collagenase and reparative collagen mimetic peptides
Source: Front Med (Lausanne). 2025 May 20;12:1514073. doi: 10.3389/fmed.2025.1514073 (PMC12131915; doi:10.3389/fmed.2025.1514073)

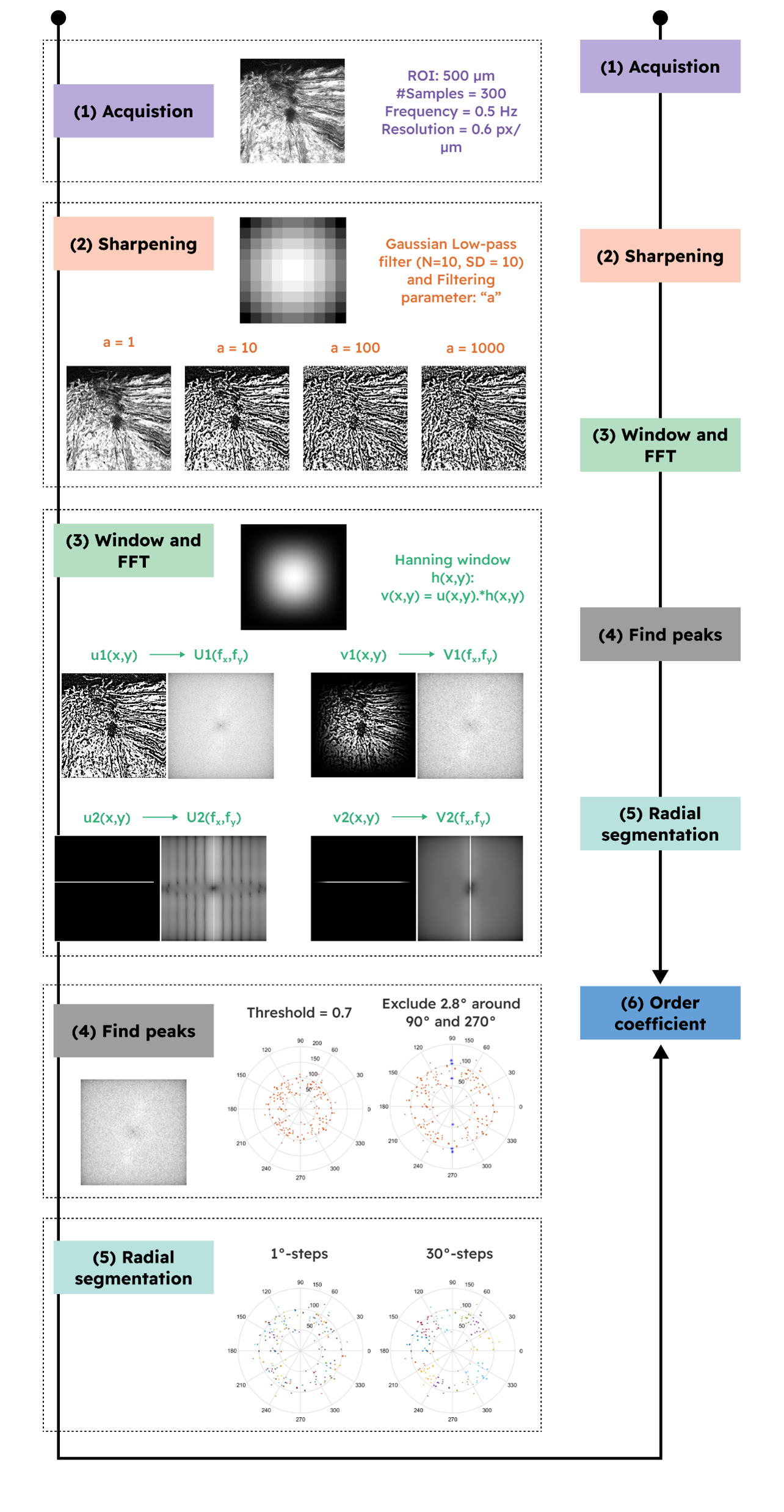

Supplement: Supplementary file 1 [file Image_1.TIF]

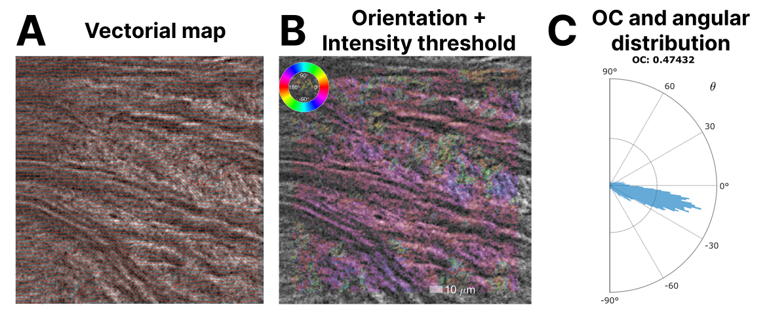

Supplement: Supplementary file 2 [file Image_2.TIF]
